# Sig2GRN: a software tool linking signaling pathway with gene regulatory network for dynamic simulation

**DOI:** 10.1186/s12918-016-0365-1

**Published:** 2016-12-23

**Authors:** Fan Zhang, Runsheng Liu, Jie Zheng

**Affiliations:** 1 School of Computer Science and Engineering, Nanyang Technological University, Singapore, 639798 Singapore; 2 Complexity Institute, Nanyang Technological University, Singapore, 637723 Singapore; 3 Genome Institute of Singapore, Agency for Science, Technology and Research, Singapore, 138672 Singapore

## Abstract

**Background:**

Linking computational models of signaling pathways to predicted cellular responses such as gene expression regulation is a major challenge in computational systems biology. In this work, we present Sig2GRN, a Cytoscape plugin that is able to simulate time-course gene expression data given the user-defined external stimuli to the signaling pathways.

**Methods:**

A generalized logical model is used in modeling the upstream signaling pathways. Then a Boolean model and a thermodynamics-based model are employed to predict the downstream changes in gene expression based on the simulated dynamics of transcription factors in signaling pathways.

**Results:**

Our empirical case studies show that the simulation of Sig2GRN can predict changes in gene expression patterns induced by DNA damage signals and drug treatments.

**Conclusions:**

As a software tool for modeling cellular dynamics, Sig2GRN can facilitate studies in systems biology by hypotheses generation and wet-lab experimental design.

Availability: http://histone.scse.ntu.edu.sg/Sig2GRN/

## Background

One of the major forms of cellular responses to extracellular perturbations is to change the gene expression in response to the cellular signals transmitted by signaling pathways. Diverse stimuli can be converted into a series of intercellular reactions through signal transduction pathways which generate various transcription factor activities, thereby producing different gene expression patterns that result in subsequent cellular behaviors.

Over the past few decades, many studies have presented various computational strategies, such as data-driven, logic-based and biochemical kinetic methods, in modeling signaling pathways or gene regulatory networks separately. Data-driven methods [1–4], which are constructed mainly based on statistical modeling, show great potential when the underlying biological mechanisms are unclear. Logic-based models, such as Boolean Network [5–11] and generalized logic models [12–16] are suitable formalisms for modeling relatively large networks in which the detailed kinetic parameters are not fully available. If the underlying biochemical mechanisms are known, biochemical kinetic modeling [17–20] is a well-established strategy for describing the dynamic sub-cellular systems using a set of mathematical equations. In the field of gene regulation, thermodynamic models have also been successfully applied [21–23] besides the aforementioned methods.

Despite the many models of signaling pathways and gene regulatory network (GRN), it is still a big challenge to link the models of signal transduction with the downstream gene expression regulation. To address this challenge, Peng et al. [24] used a set of differential equations to do forward simulations of the NF-kB signaling pathway and then used Network Component Analysis [25–27], a data-driven method based on matrix decomposition, to reversely engineer a gene regulatory network (GRN). Then they matched the forward simulations and reverse engineering results and successfully linked the signaling profiles with the subsequent gene expression profiles. However, their method needs detailed kinetic parameters which may not be available as yet in many cases. A similar study of Melas et al. [28] first employed a multi linear regression algorithm to identify correlation-based relationships between signaling proteins and cellular responses (e.g., cytokine releases) and connected them using “non-canonical” edges. Integrating a canonical network of the signaling pathway from prior knowledge, the whole network was then converted into a Boolean model. Next, they optimized the network against the experimental data using Integer Linear Programming [8] and identified the pathway activities that induced the diverse cellular responses. Their reconstructed model is able to predict the dynamics of signaling pathways and cellular responses; however, because the biological meaning of the “non-canonical” edges learnt from the data is difficult to interpret, their model can hardly reveal the molecular mechanisms of how signal transduction regulates gene expression.

Here, we present Sig2GRN, a software tool which links the models of signaling pathway with gene regulatory networks (GRNs). A generalized logical model, which we proposed previously in [13] and is based on network topology, is employed here to capture the dynamical trends of transcription factors in cellular signaling pathways. Then two different models, i.e., a Boolean model and a thermodynamics-based model [21], are integrated to predict the downstream gene expression patterns based on the predicted transcription factor activities. As a Java plugin for Cytoscape [29] (version 2.8.3), Sig2GRN is able to simulate the dynamics of the signaling pathways and the subsequent time-series gene expression data. We first provide an overview of Sig2GRN’s core functionalities, and then describe two case studies to illustrate the usage and performance of Sig2GRN.

## Methods and implementation

### Generalized logical modeling of signaling pathways for predicting transcription factor activities

**Fig. 1:**
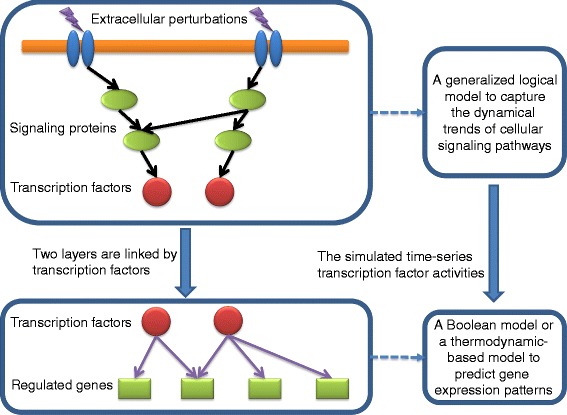
The illustration of the overall strategy. A generalized logical model is used in modeling the upstream signaling pathways to generate the activities of the transcription factors. Then the simulated transcription factor activities are used to predict the downstream gene expression according to either a Boolean model or a thermodynamic-based model

Sig2GRN takes a directed graph as the input where each vertex *x* in the network represents a molecular species (e.g., a signaling protein, a transcription factor or a gene) and each directed edge (*u*, *v*) denotes a biological interaction (e.g., protein phosphorylation or transcriptional regulation) from node *u* to node *v*. The input network is divided into two layers, i.e., the upstream signaling pathways and the downstream gene regulatory network, according to the type of biological interactions from prior knowledge (Fig. 1). The simulation starts from the user-defined perturbations and generates the dynamical trends of the signaling proteins using a generalized logical model in our previous work [13]. The goal of the upstream simulation is to generate the dynamical trends of the transcription factors under the perturbations. Then the simulated transcription factor activities are employed to predict the gene expression patterns over time, using either a Boolean model or a thermodynamic-based model [21], which can be selected by users. Therefore, the two layers of network are linked together by the transcription factors, and the cellular responses can be predicted given the extracellular perturbations.

In the upstream signaling pathways, the state *S*_*t*_ (with value ∈[0,1], where 0 means fully inhibited and 1 means fully activated) of node *s* at the *t*-th simulation iteration is updated based on its previous state at the (*t*−1)-th iteration and the incoming signals from its parent nodes, according to Eq. (1) [13],

1 $$\begin{array}{*{20}l}  {} S_{t} = & \left(1 \!- \!d\right)S_{t-1}+\left[1-\prod(1-A_{i})\right]\prod(1\,-\,B_{j})(1-S_{t-1}) \\ & -\prod(1-A_{i})\left[1-\prod(1-B_{j})\right]S_{t-1} \end{array} $$

where *A*_*i*_ (or *B*_*j*_) represents the amount of signals transmitted from the *i*-th activating (or *j*-th blocking) parent node upstream of *s*, and *d* is the degradation rate (value ∈(0,1)) at each iteration. Using this model, we have successfully predicted the dynamics of a cancer signaling pathway under various perturbations [13]. In this work, we select the simulated transcription factor activities (e.g., the proportion of the concentration of transcription factor in the active form) as the output of the upstream generalized logical mode and use them as the input to the downstream models to further predict the gene expressions as shown in Fig. 1.

### Boolean modeling of transcriptional regulation

Once the time-series data of the transcription factor activities (value ∈[0,1] at each simulation iteration) are generated, users can select either a Boolean model [30] or a thermodynamic model [21] to predict the subsequent gene expression patterns.

Under the Boolean scenario, the **AND** logical relation is assigned to the transcription factors that have the same transcriptional regulation type (e.g., activation or inhibition) for a gene, so that the gene will be switched **ON** (or **OFF**) when the maximum activity level of activating (or inhibiting) transcription factors surpasses a user-defined threshold (value ∈(0,1)). When both activation and inhibition regulations are present on the same gene, the inhibition is assumed to precede the activation. The simulation result of the Boolean model is a list of 0s and 1s, over the course of time.

### Thermodynamic modeling of transcriptional regulation

Since the Boolean simulation only refers to whether a gene up-regulated or down-regulated without revealing to what extent they will be expressed or not, we implement a thermodynamic (also termed *fractional occupancy*) model [21] to describe the gradual responses of gene expression to signal transduction. The thermodynamic model is derived under the assumption that the system is in the thermodynamic equilibrium. As such, the gene expression level is defined as a function of the activity levels of the bound transcription factors as shown in Eq. (2) [21],

2 $$\begin{array}{*{20}l}  [E]=\frac{\sum_{i \in G}\left(\prod_{j=1}^{n_{i}}K_{j}[TF_{j}]\right)Q_{i}}{\sum_{m=1}^{N}\left(\prod_{h=1}^{n_{m}}K_{h}[TF_{h}]\right)} \end{array} $$

where [ *E*] is the gene expression level, *N* is the number of all possible arrangements of transcription factors attaching to their corresponding binding sites, *G* is the set of transcription factor arrangements that turn the gene on, *n*_*i*_ (*n*_*m*_) is the number of transcription factor binding sites employed in the *i*-th (*m*-th) arrangement, *K*_*j*_ and [ *TF*_*j*_] represent the binding affinity of binding site *j* and the activity level of the transcription factor corresponding to binding site *j*, and *Q*_*i*_ is the probability of the gene being expressed when the *i*-th arrangement comprises the binding of both activating and inhibiting transcription factors (*Q*_*i*_=1 when only activating transcription factors are included).

## Results

**Fig. 2:**
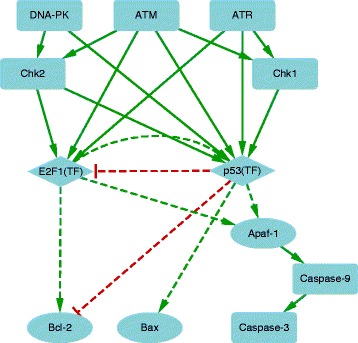
The network of cell apoptosis regulation induced by DNA damage [34]. The signals will be transmitted from the upstream signaling proteins to the transcription factors (e.g.,p53 and E2F1), then the transcription factors will regulate the transcription of apoptosis regulators (e.g., Bax, Bcl-2, Apaf-1 and Caspases). *Rectangle*, diamond and ellipse nodes represent signaling proteins, transcription factors and regulated genes, respectively. Each activation interaction is denoted as a *green edge* with an arrow head and each inhibition interaction is represented by a *red edge* with a flat-head. The *solid and dash lines* represent signal transduction and transcription regulation, respectively

**Case Study 1: DNA damage induced cell apoptosis.** DNA damage caused by ionizing radiation will activate ATM, while that by UV light will activate ATR and DNA-PK [31–33]. The stimulated kinases ATM, ATR and DNA-PK can phosphorylate p53 and E2F1 transcription factors directly or indirectly via Chk1 and Chk2. The activated p53 and E2F1 can regulate transcription of apoptosis regulator Bax, Bcl-2 and Apaf-1. Figure 2 shows the regulatory cascade of DNA damage induced apoptosis regulation. The network is constructed using GeneGo MetaCore database [34].

**Fig. 3:**
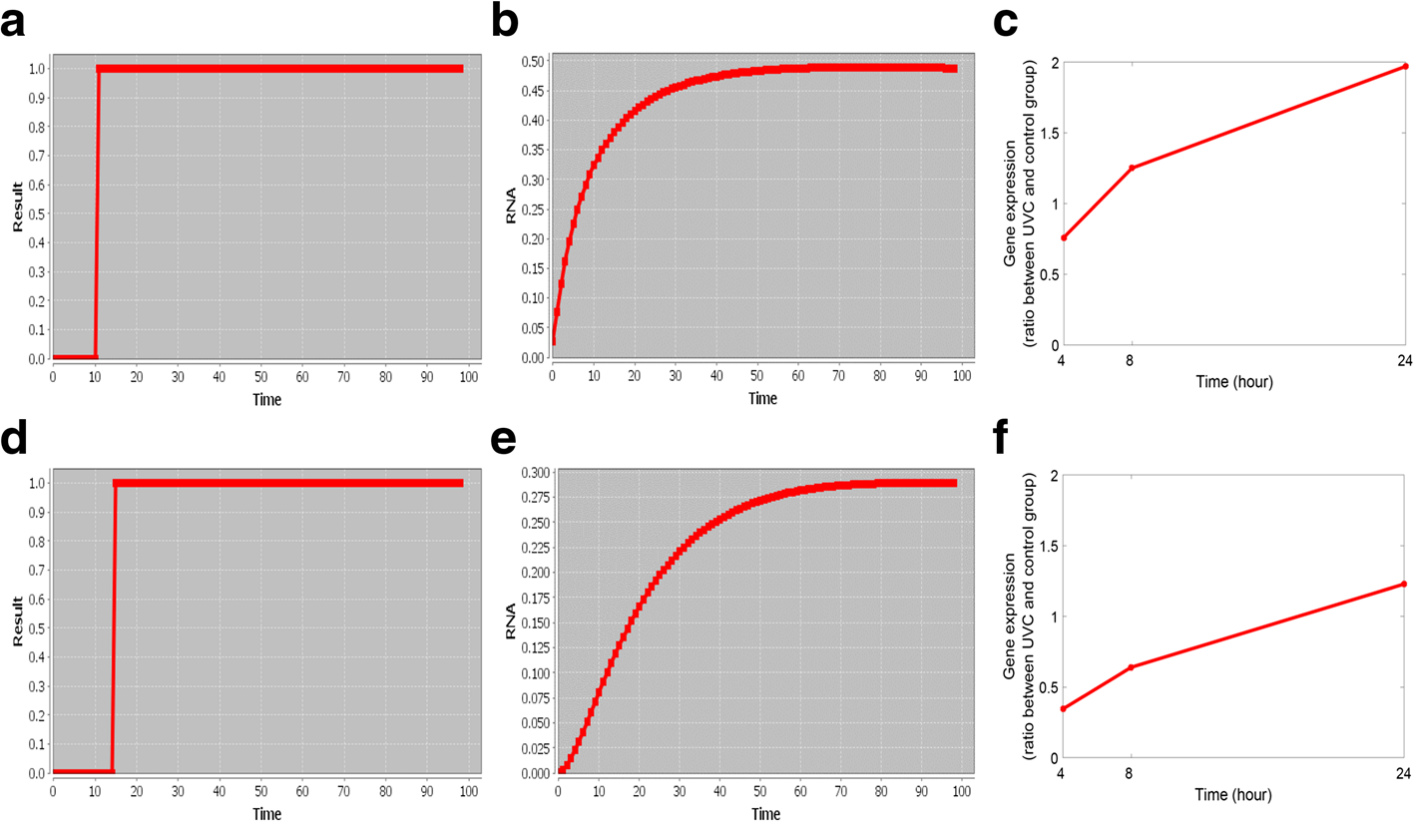
Simulated gene expression of Bax in **a** and **b** and Bcl-2 in **d** and **e** under DNA damage stimuli. ATR and DNA-PK are selected as input nodes. The parameter settings are only for illustration purpose. Both the simulated gene expression patterns of the Boolean model (plots **a** and **d**) and the thermodynamic model (plots **b** and **e**) agree with the time-series wet-lab experimental data of **c** Bax and **f** Bcl-2 [35]. The experimental data consist of the ratios of gene expression levels between UV light treated group and control group

Given the input data (value ∈[0,1]) associated with the receptors of the network (i.e., ATM, ATR and DNA-PK), the user-specified edge weights (value ∈[0,1]) and the number of iterations, Sig2GRN will first generate the dynamics of all the nodes’ activities in the network based on Eq. (1). By manually selecting the transcription factors that regulate the transcription of the genes of interest, we can run Sig2GRN to further predict this gene’s expression status over time. Figure 3 shows the simulated expression of Bax and Bcl-2 as an example. Here ATR and DNA-PK are selected as input nodes to simulate the exposure of the cells to UV light. The input levels of the input nodes were both set to 1; the edge weights of activation and inhibition interactions were set to 0.7 and 0.8, respectively; and the number of iterations was set to 100. In the Boolean model, for Bax, the selected transcription factor was p53 and the interaction type from p53 to Bax was set to activation; for Bcl-2, the transcription factors were E2F1 and p53 and the interaction types were activation and inhibition, respectively. In the thermodynamic model, the binding affinities of E2F1 and p53 were both set to 2. The parameter settings used here are only for purpose of demonstration because the prior knowledge available for parameter settings is insufficient in this case study. Moreover, the robustness of our model to the variations of the parameters (including the edge weights and the initial values of the nodes) have been empirically demonstrated in our previous work [13]. It can be seen from Fig. 3 a that Bax is expressed (in the Boolean model, 1 means the gene is expressed) after about 12 iterations when the DNA damage signals are transmitted from p53. Similar conclusion can be drawn from the thermodynamic model in Fig. 3 b that the expression of Bax increases rapidly to a plateau. In Fig. 3 d, Bcl-2 is also turned on after about 15 iterations. Compared with Bax, the simulated expression of Bcl-2 using the thermodynamic model (Fig. 3 e) increases more smoothly and the maximum expression is less than that of Bax because of incoming inhibiting signals from p53.

To validate the simulation, we use a dataset in which human TK6 cells were treated with UV light and then the gene expression was measured at three time points, i.e., 4, 8 and 24 hrs [35]. Figures 3 c and 3 f give the experimental data (the ratio of the gene expression levels between UV light treated and control groups) of Bax and Bcl-2 expression over the three time points. These two genes are the overlap between the network (Fig. 2) and the dataset [35], the dataset has measurements of many other genes which cannot be included in the network and the gene Apaf-1 in the network has no measurements in the dataset. It can be seen from the real data that the expression levels of both Bax and Bcl-2 increase over time when the cells are exposed to UV light; the slope of Bcl-2 curve is smoother and the height of the Bcl-2 curve is lower than that of the Bax curve. This suggests that, to some extent, our simulation tool is able to link the signal transduction with the gene expression regulation through transcription factors.

**Case Study 2: apoptotic signaling network treated by different combinations of drugs.** Predicting the efficacy of drugs and the design of combination therapy is a major endeavor for biomedical research and pharmaceutical industry. Lee et al. [36] studied the effects of different combinations of drugs in enhancing cell death in human breast cancer cells (cell line BT20). Here we construct a network based on their experiments and simulate the cell responses under different combinations of drug treatments to evaluate the performance of our simulator.

**Fig. 4:**
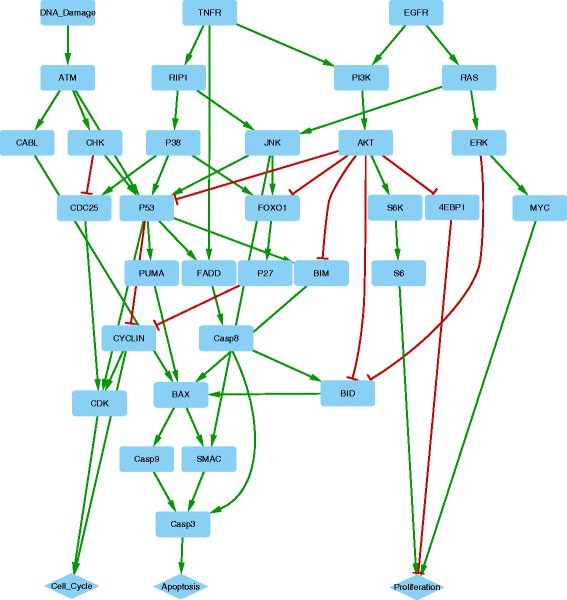
Network constructed based on [36]. Rectangle and diamond nodes represent signaling proteins and cell fates, respectively. Each activation interaction is denoted as a *green edge* with an arrow head and each inhibition interaction is represented by a *red edge* with a flat-head

The network (Fig. 4) comprises 35 nodes and 57 edges [13, 34, 36]. In the 35 nodes, 32 represent signaling proteins and 3 represent cell fates (e.g., apoptosis, proliferation and cell cycle). From the dataset in [36], we select four samples, i.e., treated with (1) EGFR inhibitor, (2) DNA damage activator, (3) both drugs and (4) the control group. The dataset has no measurement of gene expression, instead, the numbers of cells that fall into each cell fate were measured at 5 time points (i.e., 0, 6, 8, 12 and 24 hrs). Therefore, no interaction of transcriptional regulation is included in the network. We directly calculate the proportion of the dead cells at each time point as the cells response to the perturbations.

As shown in Table 1, four different types of simulation input are defined to correspond to the experimental settings in [36]. For example, half activating (0.5) signals are assigned to both TNFR and EGFR to simulate the control group; full blocking (0), half activating (0.5) and full activating (1) signals are assigned to EGFR, TNFR and DNA damage, respectively, to simulate the addition of both EGFR inhibitor and DNA damage activator together. The edge weights of activation and inhibition interactions are 0.7 and 0.8, respectively; and the number of iterations is 100. Since the network in Fig. 4 does not involve transcriptional regulation, the predicted dynamics of Caspase 3 (the only upstream node of Apoptosis) is considered as the predicted cell responses to the perturbations.

**Table 1:** Input to the simulation in case study 2

	EGFR	TNFR	DNA damage
Control	0.5	0.5	0
EGFR inhibitor	0	0.5	0
DNA damage stimuli	0.5	0.5	1
Both drugs	0	0.5	1

The columns are the input nodes in simulation and the rows are drug treatments to BT20 cells in wet-lab experiments in [36]

**Fig. 5:**
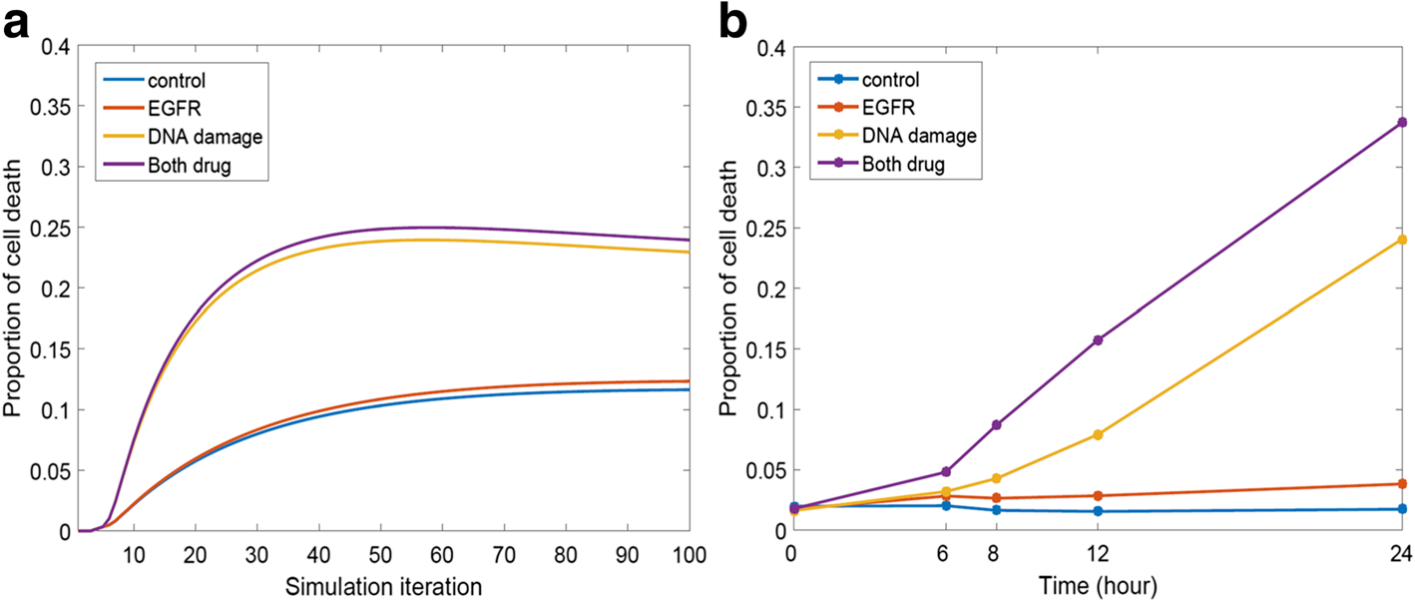
**a** Simulation results using the network in Fig. 4 and the input in Table 1. The predicted dynamics of Caspase 3 (*the only upstream node of Apoptosis*) is used as a proxy for the programmed cell death. **b** Experimental measurements of the cell death proportions under different treatments in [36]

Figure 5 a shows the simulated proportions of cell death over time. Compared with the control group (the blue curve), the addition of drugs (the orange, yellow and purple curves) enhances cell death. While the EGFR inhibitor (the orange curve) increases cell death to a small extent, the effect of DNA damage activator (the yellow curve) is significant. Furthermore, the treatment with both drugs together (the purple curve) performs the best in enhancing the cell death. Compared with the real data in Fig. 5 b, the predictions are consistent with the experimental measurements of the drug effects in terms of trends and ranking of the curves. However, there is a synergistic effect of the co-treatment in the real data, e.g., the cell death proportion induced by the co-treatment exceeds the sum of the cell death proportions induced by the two treatments separately, which has not been captured by the simulation. Moreover, mapping the simulation iterations to the real time points remains a challenge for our simulator.

## Discussion

In spite of the promising performance of our computational simulations, limitations have also been noticed. For example, in case study 2, the simulation did not reveal the synergistic effect of the co-treatment by two drugs. Possible reasons include the insufficient prior knowledge of the input networks and an oversimplification of the computational model of the nonlinear regulatory system. Moreover, since the simulation is iterated over discrete time points, it is hard to assign real time to simulation steps, which is a major obstacle for linking the two biological processes (e.g., signal transduction and transcriptional regulation) with different time scales. Techniques of multiscale modeling and simulation will be incorporated into the software in near future.

## Conclusions

Computational simulation is an important systems biology approach to the analysis of signaling pathways and gene regulatory networks. In this work, we present a software tool called Sig2GRN which is able to link the cellular signaling pathways with the downstream gene expression regulation. A generalized logical model is used in modeling the upstream signaling pathways, while a Boolean Network and a thermodynamic model are employed in modeling the downstream gene expression based on the simulated activities of transcription factors. We have shown two case studies on simulating the cell responses to the extracellular perturbations and validated the simulations with wet-lab experimental data. As a Cytoscape plugin, Sig2GRN is designed to be extensible so that more computational models of gene regulation (e.g., epigenetic modifications) can be integrated to facilitates studies in systems biology. Compared with existing methods to link signaling pathways with gene regulation, such as in [24], Sig2GRN is a parameter-free software which requires no kinetic parameters of the pathways, and thus it is still applicable when only insufficient prior knowledge of the underlying mechanisms is available. Moreover, Sig2GRN is able to predict the gene expression time-course data given the perturbations to the signaling pathways, whereas in [24] the gene expression data are required as the input of their model, which is therefore unable to predict new gene expression patterns.
